# From fragmented to integrated healthcare managing hypertension post-stroke: a qualitative study

**DOI:** 10.3389/fstro.2025.1453087

**Published:** 2025-01-30

**Authors:** Hala F. Azhari, Jonathan Hewitt, Martin O'Neill, Alexander Smith, Terence Quinn, Jesse Dawson

**Affiliations:** ^1^College of Medicine and Pharmacy, Umm Al-Qura University, Mecca, Saudi Arabia; ^2^School of Geriatric Medicine, Cardiff University, Newport, United Kingdom; ^3^Social and Economic Research Data and Methods, Cardiff University, Newport, United Kingdom; ^4^Clinical Research and Innovation Centre, St. Woolos Hospital, Newport, United Kingdom; ^5^School of Cardiovascular and Metabolic Health, University of Glasgow, Glasgow, United Kingdom

**Keywords:** stroke, hypertension, healthcare professionals, stroke survivors, public health, focus groups qualitative study

## Abstract

**Introduction:**

As stroke incidence rises with an aging population, hypertension remains a critical modifiable risk factor for both primary and secondary stroke prevention. Effective management of hypertension post-stroke requires a shift from fragmented care to integrated, patient-centered approaches. This study explores the perspectives of stroke survivors and healthcare professionals on hypertension management and evaluates the acceptability of innovative strategies, including 24-h ambulatory blood pressure monitoring (ABPM).

**Methods:**

A qualitative study using grounded theory methodology was conducted through focus group interviews with stroke survivors and healthcare professionals in Wales and Scotland between January 2019 and December 2022. Participants included 48 individuals representing diverse backgrounds and experiences. Data were analyzed thematically to identify barriers and facilitators in hypertension management post-stroke.

**Results:**

Key findings identified four major themes: the need for improved cooperation among multidisciplinary teams, knowledge gaps in stroke survivors regarding hypertension's role in stroke risk, the complexities of polypharmacy, and the potential benefits of ABPM for individualized care. Stroke survivors expressed a reliance on clinicians for hypertension management, while healthcare professionals emphasized the importance of empowering patients through education and self-management. ABPM emerged as a promising tool to enhance hypertension monitoring and support patient engagement, though practical challenges remain.

**Discussion:**

The study underscores the importance of integrating patient education, multidisciplinary care, and advanced monitoring techniques like ABPM into hypertension management. Strengthening communication pathways between patients and healthcare providers can foster greater patient engagement and accountability. Addressing socio-economic barriers, improving patient-clinician communication, and implementing holistic care strategies are critical for reducing recurrent stroke risk. These findings emphasize the need for systemic reforms and targeted interventions to bridge gaps in hypertension care delivery post-stroke.

## 1 Introduction

Stroke remains a critical public health issue worldwide, with significant morbidity and mortality implications. Globally, stroke accounts for 11.6% of all deaths and causes a staggering 143 million disability-adjusted life years lost each year (GBD 2019 Stroke Collaborators, 2021). It is estimated that approximately one in four stroke survivors will experience a recurrent stroke event (Arsava et al., 2016). Although stroke is the most prevalent secondary vascular event, stroke survivors are also at elevated risk of other ischemic events, such as myocardial infarction (Sachdeva and Ohri, 2015). While the recur is highest within the first 3 months after an initial stroke, a substantial proportion of recurrences occur years later (Sachdeva and Ohri, 2015), underscoring the ongoing need for effective secondary prevention strategies.

Among the modifiable risk factors, hypertension is especially critical in stroke prevention, as high blood pressure (BP) significantly increases the likelihood of recurrence (Makino et al., 2000). In the United Kingdom (Health Social Care Information Center, 2017), over nine million people are affected by hypertension. Lowering BP is one of the most effective measures to prevent recurrent cardiovascular events, including stroke. Despite these benefits, data from the National Health Service (NHS) Quality Outcomes Framework (Health Social Care Information Center, 2017) reveals that 16% of hypertensive patients in England still have uncontrolled BP readings above 150/90 mmHg. Furthermore, nearly 40% of patients who were on antihypertensive treatment remain uncontrolled (defined as BP above 140/90 mmHg) (Health Social Care Information Center, 2017). While intensive BP reduction to even lower targets offer enhanced vascular protection (Xie et al., 2016), achieving these targets often necessitates multiple antihypertensive drugs, potentially leading to polypharmacy and associated challenges.

Traditional single-point BP measurements, while useful, may not accurately represent an individual's BP profile across a full day. In contrast, 24-h ambulatory BP monitoring (ABPM) (Dolan et al., 2005) provides a comprehensive view of BP patterns over a full day, which is particularly valuable for assessing true BP control and more accurately predicting cardiovascular mortality risk. ABPM offers superior reliability over isolated clinic measurements by repeated BP readings outside the clinical environment, thus reflecting a more realistic BP profile for each patient (Staessen et al., 1999). However, integrating ABPM into standard clinical practice requires careful con of its acceptability and feasibility from the perspectives of both stroke survivors and healthcare providers.

This qualitative study aims to bridge the gap between current fragmented approaches and a more integrated, patient-centered model (Grover et al., 2022) for hypertension management post-stroke. By exploring the perceptions and experiences of stroke survivors and clinicians, we sought to assess the acceptability and perceived efficacy of current BP management practices as well as the potential adoption of ABPM. Insights gained from this study can inform the development of enhanced hypertension management protocols, guiding future research and policy recommendations aimed at optimizing post-stroke care. Ultimately, this work aspires to contribute to a paradigm shift in secondary prevention, addressing stroke survivors' unique needs in managing their cardiovascular health comprehensively and sustainably.

## 2 Methods

### 2.1 Study ethics

The study was conducted in accordance with the principles expressed in the Declaration of Helsinki. The study protocol was registered using the UK Integrated Research Application System (reference number: 238470) with ethical approval was obtained from the London–Harrow Research Ethics Committee with a reference number: 17/LO/2122 (NHS Health Research Authority, 2018).

### 2.2 Study design

This study employed a qualitative research design grounded in the principles of grounded theory (Glaser and Strauss, 1999), a well-established methodological framework that emphasizes the inductive development of theory from systematically analyzed data. Grounded theory is particularly suited to exploring complex social phenomena, as it enables the emergence of theory directly from participants' experiences and perspectives rather than being confined to pre-existing theoretical constructs. A constructivist approach (Stange, 2009) was chosen to guide the study data collection and interpretation processes. This approach recognizes the dynamic interaction between researchers and participants, emphasizing the co-construction of meaning. It is particularly suited to exploring the nuanced thought processes, perceptions, and experiences of stroke survivors and healthcare professionals—key elements in understanding the multifaceted challenges of hypertension management post-stroke.

The use of focus group interviews allowed for rich, interactive discussions, capturing diverse viewpoints and fostering the emergence of themes related to the barriers and facilitators of hypertension management. By prioritizing participant-driven insights, this design ensured that the findings were rooted in the lived experiences of the individuals most impacted by these issues. Furthermore, the iterative nature of grounded theory facilitated continuous refinement of emerging themes, ensuring that the analysis remained responsive to the complexity of the data. This approach not only enhanced the depth of understanding but also ensured the credibility and relevance of the study findings in addressing real-world challenges in stroke care.

### 2.3 Study approach

This study adopted a multifaceted approach to comprehensively explore the complex dynamics of hypertension management in stroke survivors. Key areas of focus included understanding participants' perspectives on effective management strategies before and after stroke and identifying significant barriers and challenges hindering optimal care. Additionally, the study examined the complex relationships among healthcare networks and stakeholders involved in hypertension management, with a particular focus on post-stroke scenarios.

Contextual and personal factors influencing hypertension management were explored in depth, including socioeconomic determinants, healthcare access, health literacy, and individual attitudes toward self-management. Both positive and negative influences were examined to provide a holistic understanding of how these factors interact to impact patient outcomes. Special attention was given to participants' perceptions of advancements in hypertension management, particularly the use of 24-h ABPM. This element was critical to understanding how ABPM could address gaps in traditional hypertension management strategies, such as the limitations of single-point BP measurements. By addressing these critical elements, the study sought to uncover both challenges and opportunities for improving hypertension management practices, fostering collaborative healthcare networks, and developing patient-centered solutions tailored to the needs of stroke survivors.

### 2.4 Focus group questions

The development of the focus group questions was a meticulous, multi-stage process, aimed at ensuring relevance, clarity, and appropriateness to the sensitive nature of the study topics. Initially, researchers developed the questions based on a thorough literature review and consultations with subject matter experts, including healthcare professionals specializing in stroke care, and qualitative research advisors. The goal was to align the questions closely with the study objectives while being sensitive to the personal experiences of stroke survivors and healthcare professionals.

Each focus group question was carefully phrased to encourage openness and allow participants to express nuanced views. The questions were designed to be open-ended, facilitating rich discussions around topics such as the collaboration within healthcare teams, the stroke survivors' understanding of hypertension and stroke etiology, and the complex social dynamics of managing polypharmacy. Additionally, questions addressed the survivors' perspectives on post-diagnosis self-management, with a particular focus on their experience with monitoring their own condition and medications.

Prior to conducting the actual focus groups, a pilot test was conducted with a small sample group representative of the main study's participants. This pilot phase helped assess the clarity, relevance, and flow of the questions, as well as the effectiveness of the topic schedule in eliciting comprehensive responses. Feedback from the pilot indicated that certain terms required rephrasing to avoid medical jargon and ensure accessibility, especially for stroke survivors who might not be familiar with specific medical terminology. Adjustments were also made to balance the pacing and sequencing of questions to enhance participant comfort and encourage a more natural flow in discussions. The pilot results also highlighted the importance of setting the right tone, which reinforced the decision to conduct focus groups in neutral venues. This choice, along with the refined question structure, contributed to creating an atmosphere that was both safe and conducive to in-depth, confidential sharing on sensitive topics.

### 2.5 Focus groups implementation

Focus groups were conducted by researchers with extensive expertise in qualitative methodology across two health board regions in Wales, UK, and one in Glasgow, Scotland. Stroke survivors with a documented history of hypertension were identified through their healthcare providers at these sites and were invited to participate in the study. Healthcare professionals directly involved in stroke and hypertension management were recruited through professional networks, specifically as members of a special interest group, ensuring the inclusion of individuals with relevant clinical expertise.

Data collection continued until thematic saturation was achieved. Saturation was determined through an iterative analysis process; whereby additional focus group sessions were assessed for emerging themes or variations within previously identified patterns. Saturation was defined as the point at which no new themes or sub-themes surfaced, and participant responses consistently aligned with prior discussions in both content and depth. This rigorous approach ensured that the data captured a comprehensive understanding of the complexities and opportunities in hypertension management post-stroke, avoiding reliance on arbitrary limits to the number of focus groups conducted.

Each focus group session was carefully documented and reviewed by the research team to ensure that thematic saturation reflected a comprehensive understanding of participants' experiences and perspectives. Through this approach, researchers confirmed that the range of viewpoints—covering variations by age, gender, socioeconomic status, and levels of disability—was sufficiently captured. The consistent recurrence of core themes, without the emergence of new insights, indicated that additional sessions were unlikely to contribute further depth to the data, affirming the point of saturation.

### 2.6 Data analysis

For data analysis, all the focus groups were digitally audio-recorded and transcribed directly for coding. One of the researchers who conducted the focus groups initially read the transcripts, listening to the recordings to correct any transcription errors and ensure accurate interpretation. The resulting transcripts were then analyzed using a thematic inductive approach (NHS Health Research Authority, 2018), which identified the key themes that emerged at each focus group.

The study themes were refined during the analysis through a systematic, iterative approach grounded in the principles of grounded theory, allowing for themes to evolve as the data were examined in depth. Initially, themes were identified using an inductive process whereby each transcript was carefully reviewed to capture emergent patterns and concepts. During this stage, the researchers applied an open coding strategy, systematically identifying a broad range of preliminary themes. As the analysis progressed, these initial codes were refined through rigorous cross-analysis, using constant comparison both within individual focus groups and across all sessions. This technique enabled the team to iteratively refine themes by comparing codes and collapsing overlapping concepts, discarding those that lacked sufficient grounding in the data or did not consistently recur across participant responses.

To enhance coherence and ensure the validity of the thematic structure, the analysis moved from open to more selective coding, aligning closely with grounded theory methodology. Themes were ultimately refined based on their relevance, coherence, and capacity to encapsulate core insights into participants' experiences. Discrepancies in theme interpretation were resolved through collaborative discussions, including triangulation with a third senior researcher, to achieve consensus on the final themes and sub-themes. This rigorous, data-driven approach ensured that the final themes were comprehensive and represented a robust synthesis of participants' perspectives, reflecting a methodologically sound application of grounded theory.

The Reporting of Qualitative Research checklist (Tong et al., 2007) criteria was followed in the reporting of this study.

## 3 Results

Stroke survivors and healthcare professionals took part in separate focus groups, provided written informed consent, and received an information sheet. Experienced qualitative researchers conducted the interviews in neutral venues, ensuring a comfortable environment detached from participants' homes or hospitals. This setting aimed to create a secure space conducive to exploring various topics ensuring confidentiality during sensitive discussions.

Between January 2019 and December 2022, a total of 48 participants were recruited for focus group discussions, including stroke survivors and healthcare professionals specializing in stroke care. One focus group consisted of stroke survivors with hypertension, evenly divided between eight females and eight males, aged between 54 and 80 years. Two additional focus groups included healthcare professionals from primary and secondary stroke care settings, comprising four stroke geriatric consultants, two endocrinologists, three occupational therapists, two stroke trainee doctors, two general practitioners, and three clinical stroke nurses. Each session, lasting ~45 min, was structured to ensure meaningful discussions, with participants grouped by their roles as either stroke survivors or healthcare professionals to facilitate targeted insights. The baseline characteristics of the study focus groups participants are detailed in Table 1.

**Table 1:** The participants baseline characteristics included in the focus groups study.

**Focus group**	**Participants' background**			
	**No**.	**Age**	**Sex**	**Baseline characteristics**
Focus group I Healthcare professionals (Wales National Health Service)	1	50	Female	Consultant geriatrician/stroke
	2	56	Male	Consultant/stroke
	3	60	Female	Consultant/stroke
	4	59	Male	Consultant geriatrician/stroke
	5	44	Male	Occupational therapist
	6	46	Male	Physiotherapist
	7	51	Female	Occupational therapist
	8	38	Male	Endocrinologist
	9	59	Female	Diabetes consultant
	10	39	Female	Trainee doctor/stroke
	11	35	Male	Trainee doctor/stroke
	12	30	Female	General practitioner
	13	33	Male	General practitioner
	14	35	Male	Nurse/stroke
	15	40	Male	Clinical stroke nurse
	16	46	Female	Nurse/stroke
Focus group II Healthcare professionals (Glasgow National Health Service)	1	50	Female	Consultant geriatrician/stroke
	2	55	Male	Consultant geriatrician
	3	46	Male	Consultant/stroke
	4	45	Female	Consultant/stroke
	5	55	Male	Occupational therapist
	6	49	Male	Occupational therapist
	7	52	Female	Occupational therapist
	8	56	Male	Diabetes specialist
	9	60	Female	Endocrinologist
	10	39	Female	Trainee doctor/stroke
	11	32	Male	Trainee doctor/stroke
	12	40	Male	Nurse/stroke
	13	42	Female	Nurse/stroke
	14	39	Female	Clinical stroke nurse
	15	35	Female	General practitioner
	16	32	Male	General practitioner
Focus group III Stroke survivors (Glasgow)	1	55	Female	Raised blood pressure, diabetes mellitus, ischemic stroke
	2	79	Male	Diabetes mellitus, raised blood pressure, ischemic stroke
	3	65	Male	Raised blood pressure, diabetes mellitus, ischemic stroke
	4	65	Male	Raised blood pressure, diabetes mellitus, ischemic stroke
	5	66	Female	Diabetes mellitus, raised blood pressure, ischemic stroke
	6	53	Female	Diabetes mellitus, ischemic stroke, raised blood pressure
	8	61	Female	Ischemic stroke, raised blood pressure, diabetes mellitus
	9	62	Male	Ischemic stroke, diabetes mellitus, raised blood pressure
	10	68	Female	Ischemic stroke, diabetes mellitus, raised blood pressure
	11	55	Male	Ischemic stroke, diabetes mellitus, raised blood pressure
	12	70	Female	Raised blood pressure, ischemic stroke, diabetes mellitus
	13	79	Male	Ischemic stroke, diabetes mellitus, raised blood pressure
	14	69	Female	Ischemic stroke, diabetes mellitus, raised blood pressure,
	15	65	Male	Ischemic stroke, diabetes mellitus, raised blood pressure
	16	50	Female	Ischemic stroke, diabetes mellitus, raised blood pressure

The analysis of these focus groups identified several interconnected themes that shaped the experiences of stroke survivors. These included the dynamics of cooperation and competition among healthcare teams in providing stroke care and aftercare, and the varying levels of knowledge and understanding among survivors regarding the relationship between hypertension and stroke risk. Participants also highlighted the challenges associated with managing polypharmacy, particularly the complexities and social dynamics of prescribing multiple medications. Additionally, the discussions emphasized the implications of post-diagnosis treatment, focusing on the role of stroke survivors in actively monitoring their condition and managing their medication regimens. Together, these findings provided a comprehensive understanding of the multifaceted challenges in post-stroke hypertension management.

### 3.1 Cooperation and competition among healthcare teams

There is an increasing recognition of the necessity for a holistic, multidisciplinary approach in managing hypertension and stroke that moves away from the traditional doctor-centered biomedical model (Foley and Timonen, 2015). Such an approach emphasizes collaboration across diverse healthcare disciplines to address the complex, multifactorial nature of these conditions. This shift requires coordination that extends beyond the purview of individual clinicians, encompassing preventive strategies for both pre-hypertension and secondary stroke prevention. Effective implementation demands the integration of services across public health, primary care, and specialist interventions, creating a framework where patients benefit from comprehensive, patient-centered care (Grover et al., 2022) rather than isolated clinical management.

A focus group participant, a secondary care physician from Glasgow, articulated the limitations of the current system with a pragmatic observation:

“*What I'd feel more comfortable with would be for people who are kind of pre-hypertension, to say, ‘Well, you've got to do some exercise. You've got to stop eating chips,' you know, because that's good for you anyway, whereas, you know, I just feel slightly cynical about guidelines that come from a pay-per-service healthcare system.”*

This perspective underscores a critical tension in contemporary healthcare. While the physician advocates for lifestyle modifications, such as dietary changes and increased physical activity, as first-line interventions for managing pre-hypertension, they also highlight the structural pressures that prioritize pharmacological treatments. These pressures, often rooted in organizational norms, reimbursement systems, and clinical guidelines, can divert focus away from addressing the root behavioral and environmental causes of hypertension.

The physician's commentary reflects broader systemic challenges, where public health initiatives aimed at prevention—emphasizing education, healthy lifestyle choices, and community-level interventions—are often under-resourced compared to the pharmaceutical-centric model of care (Marinkovic et al., 2022). Addressing this imbalance requires a paradigm shift in healthcare policy and practice, advocating for resource allocation that supports preventive care frameworks while maintaining high standards of medical treatment for individuals requiring pharmacological management. This dual focus is essential to achieve sustainable, effective outcomes in hypertension and stroke care.

### 3.2 Knowledge and awareness of hypertension and stroke etiology

Our findings reveal that stroke survivors often perceive the management of their hypertension as the primary responsibility of medical professionals, with limited recognition of their own role in actively managing the condition. Despite frequent discussions among healthcare providers about the importance of “self-management,” many stroke survivors, particularly during the pre-stroke hypertensive phase, demonstrate a lack of understanding of what self-management entails. This disconnect is reflected in their reliance on clinicians for guidance and intervention, even in cases where they had experienced prior cardiovascular events, such as heart attacks.

One participant described this disconnect candidly:

“*I had a severe heart attack 12 years ago. And from there, I was receiving medication, my BP was getting checked, and I was told it was high. So, I cannot deny that fact. But what was not thrown into the equation was if you keep it at this level, the chance of you having a stroke is increased by x amount. It wasn't put into the idiot's terms that I require so that you can say that this is going to happen if you don't do x, y, or z.”*

This testimony underscores a gap in patient education and communication. While survivors often recognize hypertension as a clinical concern, they may not fully grasp its implications for stroke risk or the actionable steps they could take to mitigate that risk. This highlights the need for clearer, more patient-centered communication strategies to empower survivors with the knowledge and tools to take a more active role in their care.

From the perspective of healthcare professionals, socioeconomic disparities further complicate hypertension management. A general practitioner from Wales highlighted these dynamics, noting differences in patient behavior across socioeconomic groups:

“*I think that's a good point about there being a difference between the sort of people who are more deprived and more affluent. I've worked in both kinds of areas, and there's no screening, but you do get more affluent people coming in a lot saying, ‘I've been checking my BP at home; it's high,' or, ‘Can I get my BP and my cholesterol checked?' whereas you don't really get that in the deprived areas, so it's definitely – there's a big sort of gap there.”*

This observation underscores the divide between what clinicians describe as the “worried well” in affluent areas—patients who seek reassurance or over-monitor their health—and the “unworried unwell” in socioeconomically deprived areas, where patients may downplay serious health concerns or adopt fatalistic attitudes. Such attitudes often result in poorer adherence to treatment regimens and lower engagement in preventative health behaviors, perpetuating health inequities.

These findings highlight the interplay between patient perceptions, socioeconomic context, and healthcare communication in hypertension management. Bridging these gaps requires targeted educational interventions that are sensitive to socioeconomic realities, as well as systemic efforts to enhance access to preventative care for underserved populations. By addressing these disparities, healthcare providers can foster greater patient engagement and improve outcomes in hypertension and stroke prevention.

### 3.3 Polypharmacy and prescribing dynamics

Stroke survivors often face significant challenges related to polypharmacy due to the complexity of their comorbidities, which necessitates consulting multiple doctors and specialists. This fragmented care frequently results in the prescription of numerous medications, leaving many survivors feeling confused and overwhelmed. A common concern among participants was the lack of understanding regarding the purpose of each medication and the potential interactions between them. One stroke survivor from Wales described this sense of unease:

“*Basically (I'm) on a pile of drugs that interact with each other, and as I was told when I ask the question, nobody really knows what one drug's doing to the other. When you're on such a mixture, that can be a difficulty.”*

This statement highlights the anxiety and lack of control many stroke survivors feel about their treatment regimens. The perception that even healthcare professionals may not fully understand the cumulative effects of multiple medications exacerbates these concerns. Survivors also expressed a sense of powerlessness in navigating the hierarchical dynamics of medical care. As one participant from Glasgow shared:

“*You can go and see your GP and ask him why he's put you on that medication. But it's a brave GP that turns around and says, ‘You don't need it,' when your consultant cardiologist has put you on x, y, and z… And then the pharmacist comes in and says, ‘You really shouldn't be taking those two together.' You've got to trust the professionals.”*

This testimony underscores the complex interplay of authority, trust, and communication in polypharmacy management. Stroke survivors often feel caught between conflicting recommendations from different healthcare providers, leading to confusion and frustration. These experiences point to a broader systemic issue where the lack of coordinated care can undermine patients' confidence in their treatment.

From the perspective of healthcare professionals, the issue of polypharmacy raises critical questions about clinical guidelines and their applicability to certain patient populations, particularly the frail older adult. A secondary care physician from Glasgow articulated concerns about the appropriateness of aggressive antihypertensive treatment for this demographic:

“*In terms of absolute risk, the people who are at greatest risk are frail older adult, but I just wonder what we're trying to achieve. In someone who's only got a life expectancy of, say, a year regardless of what you do, do you really want to be hammering them with fixed antihypertensives to get them to a BP threshold that's slightly arbitrary in that group? So, all of that wouldn't apply to, you know, someone in their 40 s with quasi-hypertension plus renal failure plus diabetes, but I think the group that we see in secondary care are a comorbid, frail group of people with very limited life expectancy, and I just wonder if we're doing the right thing by hammering them to target.”*

This perspective highlights the ethical and clinical dilemmas in balancing guideline-directed care with individualized treatment. While younger patients with multiple risk factors might benefit from strict BP control, aggressively targeting BP in frail older adult patients with limited life expectancy may provide little benefit and could introduce additional risks.

These insights reveal the multifaceted challenges of managing polypharmacy in stroke survivors, particularly the need for a patient-centered approach that prioritizes clear communication, shared decision-making, and the careful evaluation of treatment goals. Coordinated care strategies, such as regular multidisciplinary medication reviews and enhanced collaboration among healthcare providers, could help address these issues. Such interventions are essential for reducing the burden of polypharmacy and improving the quality of care for stroke survivors across diverse clinical and demographic contexts.

### 3.4 Post-diagnosis treatment implications

The clinician has extensive hands-on experience with the use of 24-h BP monitoring devices, which not only enhances the engagement of stroke survivors in their healthcare management but also introduces a more comprehensive and dynamic approach to BP monitoring. This approach, they believe, could play a significant role in encouraging patients to take a more proactive stance in managing their hypertension. As a primary care physician, they explain the variations across different healthcare settings:

“*I have worked across several health boards, each with its own unique setup. For example, in one practice, they had a substantial number of BP monitors available, and it was a standard procedure to send patients home with one. They would be instructed to record their BP multiple times a day over the course of a week and then bring the data back to discuss with me. In contrast, at my current practice, we don't have access to that particular model, but we do have easy access to 24-h ambulatory BP monitoring. When we encounter patients with very high BP, we initiate treatment immediately. However, for those with borderline or slightly elevated readings, we often prefer to start with the 24-h monitoring as it provides more accurate, continuous data, allowing for better clinical decision-making.”*

The clinician further emphasizes how this approach fosters a deeper understanding of patients' BP patterns, which helps tailor a more individualized treatment plan:

“*I firmly believe that providing patients with home BP monitors could significantly enhance the management of their condition, especially after hospital discharge or post-clinic visits. I feel much more confident and proactive in managing a patient's hypertension when they bring in a detailed log of BP readings they've recorded over the past 2 weeks. Having that continuous data is far more reliable than relying on a single, isolated reading, which often leaves a lingering doubt in the back of my mind about its accuracy or representativeness of the patient's true condition.”*

This approach not only empowers stroke survivors to actively participate in their own health management but also provides clinicians with a clearer, more comprehensive picture of their BP trends, leading to more informed, targeted interventions.

**Figure 1:**
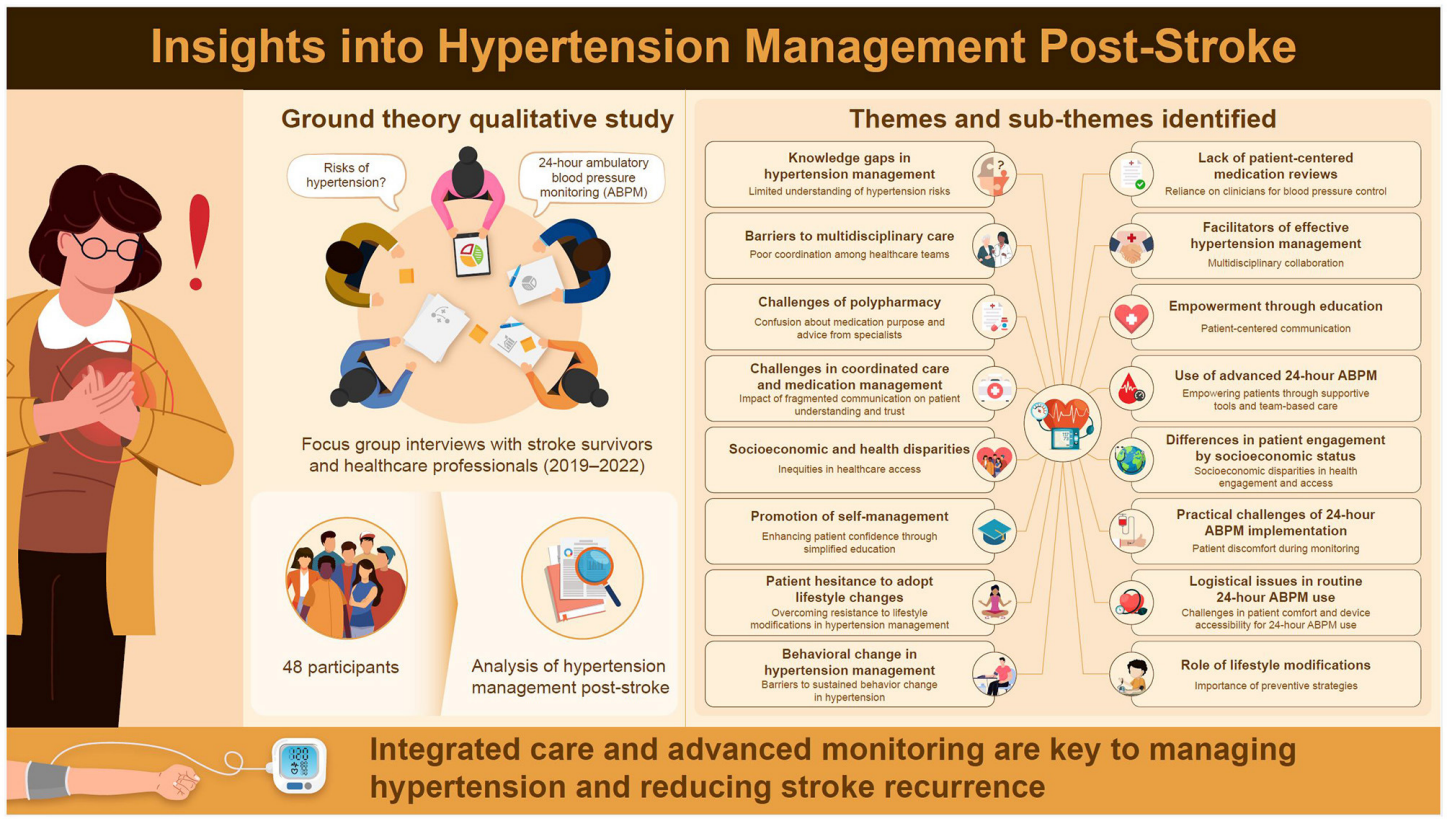
Insights into hypertension post-stroke.

Expanding the thematic analysis involved synthesizing participant responses into clearly defined categories that captured the essence of their experiences while aligning with the study's objectives. By grouping related insights under broader, well-articulated themes, the analysis gained depth and coherence. Participant responses were categorized based on recurring challenges, such as knowledge gaps and system-level barriers, as well as facilitators, including effective multidisciplinary collaboration and patient education. Additionally, these categories were integrated with relevant theoretical frameworks, such as the patient-centered care model (Grover et al., 2022), the doctor-centered biomedical model (Foley and Timonen, 2015), and the pharmaceutical-centric model of care (Marinkovic et al., 2022). These frameworks provided a comprehensive perspective on the data, contextualizing participant responses and linking individual behaviors and perceptions to broader psychosocial or systemic factors. This approach not only enriched the understanding of participant experiences but also highlighted critical areas for intervention and improvement in post-stroke hypertension management, as detailed in Table 2 and Figure 1.

**Table 2:** The expanded thematic and sub-thematic analysis of the barriers and facilitators in post-stroke hypertension management.

**Themes**	**Sub-theme(s)**	**Participant Insights**	**Integration with theoretical frameworks**
Knowledge gaps in hypertension management	Limited understanding of hypertension risks	“*I wasn't told how keeping blood pressure at this level increases the chance of stroke.”*	Health belief and patient-centered care model: Highlights how low perceived severity and inadequate cues to action hinder proactive patient involvement. Suggests interventions to improve patient education on hypertension's role in stroke
Barriers to multidisciplinary care	Poor coordination among healthcare teams	“*Doctors manage my condition; I just follow their advice.”*	
Challenges of polypharmacy	Confusion about medication purpose and advice from specialists	“*Sometimes one doctor says one thing, and another says the opposite.”*	Systems theory and pharmaceutical-centric model of care: Emphasizes the necessity of integrated care frameworks to improve communication and streamline patient management across multiple disciplines.
Challenges in coordinated care and medication management	Impact of fragmented communication on patient understanding and trust	“*I don't feel there's a single person coordinating my care.”*	
Socioeconomic and health disparities	Inequities in healthcare access	“*I need clear guidance on how to check my blood pressure and what changes to make.”*	Self-efficacy theory and patient-centered care model: Focuses on building confidence through education and training, empowering patients to take active roles in managing their hypertension.
Promotion of self-management	Enhancing patient confidence through simplified education	“*If someone explained it in simple terms, I'd know what to do.”*	
Patient hesitance to adopt lifestyle changes	Overcoming resistance to lifestyle modifications in hypertension management	“*Nobody told me that exercise and diet are as important as medication.”*	Transtheoretical of change and patient-centered care model: Focuses on guiding patients through stages of change to adopt and sustain healthier behaviors as part of hypertension management.
Behavioral change in hypertension management	Barriers to sustained behavior change in hypertension	“*I know I should, but it's hard to stay motivated for long.”*	
Lack of patient-centered medication reviews	Reliance on clinicians for blood pressure control	“*I'm on so many drugs; it's hard to know what they're all for.”*	Shared decision-making and doctor-centered biomedical model: Advocates for collaborative approaches in medication management, involving patients in discussions to reduce confusion and enhance treatment adherence.
Facilitators of effective hypertension management	Multidisciplinary collaboration	“*I feel nobody truly understands how all these medications interact.”*	
Empowerment through education	Patient-centered communication	“*24-h ambulatory blood pressure monitoring helped me see my blood pressure trends better than a single clinic reading.”*	Theory of planned behavior and patient-centered care model: Highlights how tools like 24-h ambulatory blood pressure monitoring improve perceived behavioral control and patient engagement in hypertension self-management.
Use of advanced 24-h ambulatory blood pressure monitoring	Empowering patients through supportive tools and team-based care	“*Having a team made me feel more supported in managing my condition.”*	
Differences in patient engagement by socioeconomic status	Socioeconomic disparities in health engagement and access	“*Affluent patients ask about their blood pressure; in deprived areas, people don't come forward until it's too late.”*	Social determinants of health and doctor-centered biomedical model: Explores how socioeconomic factors influence health behaviors and outcomes, emphasizing targeted interventions to reduce disparities.
Practical challenges of 24-h ambulatory blood pressure monitoring implementation	Patient discomfort during monitoring	“*Not everyone has the resources to manage their health properly.”*	
Logistical issues in routine 24-h ambulatory blood pressure monitoring use	Challenges in patient comfort and device accessibility for 24-h ambulatory blood pressure monitoring use	“*The device was uncomfortable to sleep with.”*	Technology acceptance and doctor-centered biomedical model: Addresses the need for improving usability and accessibility of 24-h ambulatory blood pressure monitoring to enhance its adoption in clinical settings and patient satisfaction.
Role of lifestyle modifications	Importance of preventive strategies	“*Not every clinic can afford or manage 24-h ambulatory blood pressure monitoring for all patients.”*	

## 4 Discussion

This study aimed to understand the perspectives of stroke survivors and healthcare professionals on BP management post-stroke, with a particular focus on the acceptability and perceived efficacy of 24-h ABPM compared to traditional single clinic measurements. The study highlights significant gaps and challenges in managing hypertension post-stroke, emphasizing the need to transition from fragmented to integrated healthcare models to optimize outcomes for stroke survivors. Stroke survivors and healthcare professionals alike recognize the essential roles played by various healthcare specialties in comprehensive hypertension management. However, achieving a cohesive, multidisciplinary approach is often impeded by differing organizational priorities, funding limitations, and variations in institutional culture. These barriers can result in fragmented care, where the complexity of stroke patients' needs—particularly regarding comorbid conditions like hypertension—is not fully addressed.

A comprehensive, team-based approach to hypertension management could bridge traditional, provider-centered care (Tong et al., 2007) with the growing need for patient-centered (Grover et al., 2022), holistic models. This approach would combine pharmacological treatment with lifestyle interventions tailored to each patient's unique health needs and socioeconomic factors. Such a model aligns with broader healthcare trends toward personalized care that considers social determinants of health and disparities in access. Specific strategies might include structured, multidisciplinary educational sessions that clarify the hypertension-stroke link in accessible language, using visual aids, real-life scenarios, and interactive components to reinforce learning. Digital health tools—such as BP tracking apps with embedded educational modules—could further support continuous patient engagement, offering real-time insights into BP patterns alongside prompts on lifestyle modifications, medication adherence, and BP management. Follow-up consultations with nurses or health coaches (Zuo et al., 2019) could provide ongoing support, reinforcing key messages and offering individualized guidance. Collectively, these interventions would promote a proactive, patient-centered approach to hypertension management, empowering stroke survivors to actively participate in strategies for BP control and stroke risk reduction.

Many stroke survivors perceive BP control as primarily the responsibility of healthcare providers, revealing a critical need for targeted educational efforts to empower patients. Enhancing patient understanding of hypertension's impact on stroke recurrence can foster greater engagement in self-management (Bosworth et al., 2010), which is crucial for long-term BP control and improved outcomes. Effective educational interventions (Boden-Albala and Quarles, 2013) must extend beyond simple information sharing, actively involving stroke survivors in recognizing the essential role of BP management in reducing their risk of recurrence.

Concerns around polypharmacy and the complexity of medication regimens are particularly significant for stroke survivors, who often feel a lack of control over their treatment and experience challenges with self-management. These concerns are further exacerbated by fragmented communication and inconsistent coordination among healthcare providers, frequently leaving patients with incomplete or conflicting information. Addressing these issues requires stewardship implementing strategies (Daunt et al., 2023) to manage polypharmacy more effectively in clinical practice. One approach is the development of individualized medication review sessions, where pharmacists or multidisciplinary care teams collaborate with patients to review each medication's role, benefits, and potential interactions. These sessions could empower patients by improving their understanding of their regimens and addressing any misconceptions. A second strategy involves establishing structured communication pathways, such as shared electronic health records accessible to all involved providers, to ensure consistent, accurate medication information across specialties. Additionally, incorporating shared decision-making (US Preventive Services Task Force, 2022) practices into clinical encounters is essential; clinicians can engage patients in discussing the goals, risks, and potential side effects of their medications. For patients with complex comorbidities, medication reconciliation at each point of care transition—such as hospital discharge or specialist referrals—can help prevent unnecessary prescriptions and simplify regimens. These strategies not only aim to streamline polypharmacy but also foster a sense of partnership in care, enhancing patients' confidence in managing their health post-stroke.

Although hypertension remains a primary modifiable risk factor for recurrent stroke, yet a range of barriers hinders effective BP control and risk reduction in this population. Despite advances in pharmacological therapy, a substantial proportion of hypertensive patients continue to have elevated BP readings, with many individuals on treatment still not achieving target BP control (Health Social Care Information Center, 2017). This highlights an urgent need for improved monitoring strategies that offer a more complete and accurate assessment of BP patterns, which could significantly enhance hypertension management precision and reduce the risk of recurrent strokes in vulnerable populations.

Current guidelines (Cheung et al., 2023) and clinical practices often rely on isolated BP measurements taken in clinical settings, which may not accurately reflect an individual's true BP profile across diverse daily conditions and nocturnal periods. The integration of 24-h ABPM (Dolan et al., 2005) represents a promising strategy to address these challenges in hypertension management post-stroke. ABPM provides a more accurate assessment of BP fluctuations across a full day, capturing variability and nighttime surges that are often missed by isolated clinic readings. This comprehensive monitoring approach could enhance secondary stroke prevention by offering actionable insights that support personalized antihypertensive regimens. However, practical considerations must be addressed, as patients reported challenges with the physical demands of ABPM, particularly during nighttime hours, and clinicians noted logistical constraints in routinely implementing this tool. Addressing these concerns will require tailored strategies, including patient education on ABPM's value (Lee, 2024), to improve adherence, as well as healthcare policy adjustments to allocate resources effectively for ABPM implementation.

Strategically integrating ABPM into routine post-stroke care would necessitate a multifaceted plan. This includes revising national and regional guidelines to establish ABPM as a standard protocol for BP monitoring in high-risk stroke survivors, particularly those with uncontrolled hypertension or notable BP variability. Policy support should focus on funding and infrastructure investments to facilitate ABPM access within healthcare settings. Additionally, training programs for healthcare professionals on the interpretation and application of ABPM data are crucial to foster a nuanced approach to BP management. Patient education initiatives should also be developed to mitigate potential discomfort associated with ABPM and promote adherence, as patient engagement is fundamental to the success of this approach. Together, these strategies could strengthen the integration of ABPM into clinical practice, ultimately supporting more precise and individualized BP control.

In light of previous research on post-stroke comorbidity management, our findings align with study on post-stroke diabetes and hypertension management (Hewitt et al., 2024) and the COMPOSEd study (Azhari et al., 2024), which emphasize the importance of coordinated, patient-centered care for stroke survivors with multiple chronic conditions. Such studies underscore the value of integrating medical management with patient education, self-management strategies, and enhanced healthcare coordination. This integrative approach is essential for addressing the complexities of hypertension management post-stroke, fostering a healthcare model that improves overall care outcomes and empowers patients within an accessible, supportive framework.

### 4.1 Implications for future research

Although prior studies have highlighted the benefits of self-management (Fugazzaro et al., 2021) for stroke survivors with hypertension, the unique challenges faced by pre- and post-stroke individuals suggest that a one-size-fits-all approach is insufficient. A genuinely patient-centered strategy should be developed to support hypertension self-management in both primary and secondary stroke prevention contexts. This approach must address not only the negative experiences (Srivastava et al., 2010) of patients—such as fears, depression, and fatigue—but also their aspirations and goals, recognizing that these psychological factors are integral to the effectiveness of self-management interventions.

Empowering stroke survivors with hypertension to effectively self-manage their condition necessitates a multifaceted strategy that integrates personalized education (Collins et al., 2017). Personalized education programs should be designed to address the specific needs and knowledge gaps of stroke survivors, using simple, relatable language to explain the link between hypertension and stroke. To implement this, healthcare providers should consider tailored education programs that address specific knowledge gaps in stroke survivors, using accessible language and culturally sensitive materials in various formats, such as written guides, videos, and interactive digital modules. Hands-on workshops could equip patients with practical skills for BP monitoring, medication management, and lifestyle changes such as diet and exercise. The integration of digital health tools, including mobile applications and wearable devices, would further support real-time monitoring and encourage patient engagement. Additionally, telehealth services and online support communities (Foroumandi et al., 2020) can provide continuous support and motivation, helping to mitigate feelings of isolation.

Multidisciplinary support is critical in fostering a sustainable self-management framework. A coordinated stroke care team—including physicians, nurses, pharmacists, dietitians, occupational therapists, and social workers—can deliver consistent, comprehensive care, with family involvement reinforcing the support network. Addressing psychological barriers is also essential; mental health support (Lorig et al., 2001) can alleviate depression and anxiety, which are common post-stroke and can hinder self-management. Regular follow-ups and adaptive feedback mechanisms will help keep patients aligned with their goals, while addressing socioeconomic barriers (McManus et al., 2018) through community resources and financial assistance can make self-management accessible across diverse patient populations. Implementing these recommendations would not only address immediate needs but also underscore the necessity for further research to evaluate their long-term impact on health outcomes and stroke recurrence.

### 4.2 Strengths and limitations

The study applies a theoretical framework for acceptability (Sekhon et al., 2017) by integrating perspectives from healthcare professionals and stroke survivors to explore the feasibility of ABPM as a self-management tool in stroke survivors. This approach combines thematic analysis with focus groups to facilitate in-depth discussions among key stakeholders. While efforts were made to allow all participants equal opportunities to respond, the study may be subject to dominant respondent bias (Brandner and Hood, 2021), where certain participants may have monopolized discussions, potentially influencing the responses of others. Additionally, while healthcare professionals were recruited from primary and secondary stroke centers in Glasgow and Wales to encourage diversity in clinical experience, social desirability bias (Larson, 2019) remains a concern. Some healthcare professionals may have responded in ways they believed aligned with perceived expectations, rather than fully reflecting their clinical practice.

The purposive sampling strategy aimed to capture a range of perspectives on hypertension management post-stroke, leveraging a qualitative approach to gain insights into diverse viewpoints. However, this sampling within localized healthcare settings limits the transferability of findings to other cultural or geographic contexts, and the small sample size restricts the generalizability of results beyond the specific populations studied. Although theoretical saturation (Mason, 2010) was reached, indicating that the themes identified are robust within the sample, the limited diversity and scale of the sample suggest that future studies could benefit from larger, more diverse samples across different settings to validate these findings.

Furthermore, the study's qualitative design inherently restricts quantitative measures of effect, and additional longitudinal or mixed-method studies may help to substantiate the themes identified here and assess their broader relevance. Future research should address these limitations by including larger, more heterogeneous samples, conducting studies in varied cultural and healthcare settings, and integrating quantitative measures to support a more comprehensive understanding of ABPM's acceptability and effectiveness in stroke self-management. Addressing these limitations would enhance the generalizability and applicability of findings, ultimately strengthening the evidence base for hypertension self-management strategies in stroke care.

## 5 Conclusion

This study highlights the challenges of hypertension management post-stroke and emphasizes the need for an integrated, patient-centered approach to optimize outcomes. Fragmented healthcare systems, knowledge gaps among stroke survivors, and the complexities of polypharmacy create significant barriers to effective care. Addressing these issues requires a multidisciplinary strategy that incorporates education, self-management support, and innovative monitoring tools such as ABPM. Empowering stroke survivors through tailored educational interventions and involving them in the management of their condition are critical for long-term success. Additionally, adopting holistic approaches that account for socio-economic disparities and enhance patient engagement can bridge gaps in care and promote adherence to treatment plans. Future research should explore scalable strategies to integrate these elements into standard practice, ultimately advancing secondary prevention efforts and improving the quality of life for stroke survivors with hypertension.

## Data Availability

The original contributions presented in the study are included in the article/supplementary material, further inquiries can be directed to the corresponding author.
